# From Evidence to Practice: A Narrative Framework for Integrating the Mediterranean Diet into Inflammatory Bowel Disease Management

**DOI:** 10.3390/nu17030470

**Published:** 2025-01-28

**Authors:** Riya Gautam Naik, Sarah A. Purcell, Stephanie L. Gold, Victoria Christiansen, Leah D. D’Aloisio, Maitreyi Raman, Natasha Haskey

**Affiliations:** 1 Department of Biology, Irving K. Barber Faculty of Science, University of British Columbia Okanagan, Kelowna, BC V1V 1V7, Canada; riyagn@student.ubc.ca (R.G.N.); leahdaloisio@hotmail.com (L.D.D.); 2 Centre for Chronic Disease Prevention and Management, Southern Medical Program, Faculty of Medicine, University of British Columbia, Kelowna, BC V1V 1V7, Canada; sarah.purcell@ubc.ca; 3 Division of Endocrinology, Department of Medicine, University of British Columbia, Vancouver, BC V5Z 1M9, Canada; 4 School of Health and Exercise Sciences, Faculty of Health and Social Development, University of British Columbia Okanagan, Kelowna, BC V1V 1V7, Canada; 5 The Henry D. Janowitz Division of Gastroenterology, Icahn School of Medicine at Mount Sinai, New York, NY 10029, USA; stephanie.gold@mountsinai.org; 6 Gastroenterology Clinic, Red Deer Regional Hospital, Department of Nutrition Services, Alberta Health Services, Red Deer, AB T4N 4E7, Canada; victoria.christiansen@albertahealthservices.ca; 7 Division of Gastroenterology and Hepatology, Department of Medicine, Cumming School of Medicine, University of Calgary, Calgary, AB T2N 1N4, Canada; mkothand@ucalgary.ca

**Keywords:** Mediterranean diet, ulcerative colitis, Crohn’s disease, nutrition therapy

## Abstract

Emerging evidence underscores the pivotal role of diet in preventing and managing inflammatory bowel disease (IBD). As our comprehension of the microbiome’s role in IBD expands, dietary modifications are increasingly recognized as potential adjuncts or primary therapeutic strategies. Key components of the Mediterranean diet (MD)—including microbiota-accessible carbohydrates, omega-3 fatty acids, polyphenols, and antioxidants—have demonstrated promise in enhancing gut microbiota diversity and reducing intestinal inflammation, making it a practical approach for managing IBD. Moreover, the MD offers additional benefits considering the rising prevalence of comorbid chronic inflammatory conditions such as diabetes, cardiovascular disease, and obesity in IBD patients. The purpose of this narrative review was to provide an overview of the feasibility and clinical outcomes of the MD and offer evidence-based guidance for researchers and practitioners on how to adapt the MD to patients with IBD. According to several cross-sectional and interventional studies, the MD is feasible for patients with IBD and confers several benefits, such as reduced inflammation, improved disease activity, and enhanced quality of life, with a strong adherence rate and minimal adverse effects. To facilitate knowledge translation, we provide a practical framework for integrating the MD as a nutritional therapy for IBD, including specific recommendations and messaging that researchers, practitioners, and patients can use. By synthesizing current evidence and offering actionable insights, the aim is to facilitate the integration of the MD into IBD management, with the potential to improve patient outcomes.

## 1. Introduction

Inflammatory bowel disease (IBD), which includes Crohn’s disease (CD) and ulcerative colitis (UC), is a chronic, debilitating inflammatory condition affecting the gastrointestinal tract. Its relapsing and remitting pattern, characterized by symptoms such as diarrhea, abdominal pain, rectal bleeding, and weight loss, negatively impacts patients’ morbidity and quality of life [1]. While advancements in treatments have provided relief for some, a “therapeutic ceiling” has been reached, as 30–40% of patients do not respond or lose response to biological therapies [2,3]. This challenge has ignited growing interest among patients and clinicians in non-pharmacological strategies, particularly dietary interventions, which promise to reshape the gut microbiota, restore microbial balance, and reduce intestinal inflammation.

The increasing global prevalence of IBD, particularly in newly industrialized regions such as South America, Eastern Europe, South Asia, and Africa, as well as among immigrants transitioning from developing to developed countries, highlights the critical role of environmental factors in the pathophysiology of the disease [4]. The increase in IBD cases among ethnic groups and nationalities, where it was previously uncommon, is closely linked to the adoption of the Western lifestyle [5]. This results in alterations in environmental factors, including better hygiene standards, lifestyle shifts, changes in nutritional patterns, and modifications in food products, including increased quantities of xenobiotics in food [6]. The Western diet of today is characterized by a high consumption of refined sugars, refined carbohydrates, sodium, animal proteins, and ultra-processed foods, which contrasts sharply with the traditional diets of previous generations [7]. Growing evidence highlights the detrimental impact of this modern dietary pattern on the gut microbiome [8]. Alterations in the microbiota compromise intestinal barrier integrity, allowing antigenic microbial and diet-derived components to translocate into the underlying mucosa [7]. This triggers an abnormal immune response and perpetuates a cycle of inflammation, further linking dietary factors to the onset and worsening of chronic conditions such as IBD [8].

The Mediterranean diet (MD) is a whole-food, plant-based dietary approach recommended for patients with IBD [9]. Its core elements include high consumption of olive oil and plant-based foods such as vegetables, fruits, whole grains, legumes, nuts, and seeds, moderate intake of fish, seafood, and dairy, low-to-moderate alcohol consumption (primarily red wine), and limited intake of red meat and processed foods [10]. The MD emphasizes unprocessed, anti-inflammatory foods, promoting a diet rich in microbiota-accessible carbohydrates, lean protein, and omega-3 fatty acids [10]. Due to its established benefits in enhancing gut microbiota diversity, composition, and function, as well as its anti-inflammatory properties—both in healthy individuals and emerging studies in IBD [11,12,13,14]—the best practice guidelines recommend that all patients with IBD should be encouraged to adopt the MD for its potential to improve gut health and manage inflammation [9].

Several studies support the importance of diet in the management of IBD, with a growing number of solid food diets for patients with IBD [15,16]. A comprehensive comparison of these approaches is beyond the scope of this review; however, exclusive enteral nutrition, the Crohn’s disease exclusion diet (CDED), the specific carbohydrate diet (SCD), and the low fermentable oligosaccharides, disaccharides, monosaccharides, and polyol (FODMAP) diet show promise for improving the management of IBD [17]. Exclusion diets have been the primary focus for managing IBD, with few studies exploring the benefits of inclusion diets, such as the MD [15,18,19]. This shift in perspective emphasizes incorporating nutrient-rich, health-promoting foods rather than focusing on elimination. This narrative review critically examines the unique attributes and potential benefits of the MD in IBD management. By synthesizing the existing literature, this review aims to highlight the MD’s effects on IBD outcomes and offer practical guidance for integrating this dietary approach in to clinical practice.

## 2. Methods

To enhance the rigor of this narrative review, a literature search was conducted in PubMed from inception until 4 June 2024 to identify English-language articles involving human participants. To identify relevant articles, we combined IBD-related terms ((Inflammatory Bowel Diseases[mh] OR “inflammatory bowel disease*” [Title/Abstract] OR IBD[Title/Abstract] OR Crohn*[Title/Abstract] OR “ulcerative colitis” [Title/Abstract])) with MD-related terms (“Mediterranean diet” [Title/Abstract] OR Mediterranean diet [mh]). The reference lists of articles identified on PubMed were also reviewed to identify other potentially relevant articles. Both observational and interventional studies were included.

## 3. The Mechanisms for Diet in Inflammatory Bowel Disease

The bacteriome in IBD is altered and is characterized by the loss of beneficial microbes, expansion of pathobionts, and reduced microbial diversity [20]. It is well established that patients with IBD have reduced levels of beneficial anaerobic microbes, including *Faecalibacterium prausnitzii*, *Roseburia, Bacteroides*, *Suterella*, *Bifidobacterium*, and *Lachnospiraceae* [20]. These microbes play a critical role in breaking down microbiota-accessible carbohydrates (MACs) to produce short-chain fatty acids (SCFAs) like butyrate, acetate, and propionate. SCFAs are highly relevant in IBD due to their ability to modulate immune responses and maintain intestinal homeostasis [21]. They reprogram the metabolism of innate immune cells like macrophages, monocytes, and neutrophils, promoting anti-inflammatory phenotypes and reducing pro-inflammatory cytokine production. SCFAs inhibit histone deacetylases (HDACs), leading to epigenetic suppression of pro-inflammatory genes, and they downregulate nuclear factor-kappa B (NF-κB) signaling, a central driver of inflammation in IBD [21,22]. Additionally, SCFAs strengthen the intestinal barrier by enhancing epithelial cell function, mucin production, and tight junction integrity, preventing microbial translocation and excessive immune activation [21]. The alterations in SCFAs in IBD, often linked to microbial dysbiosis and low MAC intake, contribute to disease pathogenesis, highlighting their therapeutic potential [23,24].

In contrast, patients with IBD have an overgrowth of bacteria such as *Escherichia*, *Clostridioides difficile*, *Salmonella*, *Enterobacteriaceae*, and *Proteobacteria* [20]. These microbes can disrupt gut homeostasis by producing pro-inflammatory metabolites, such as lipopolysaccharides (LPS), that stimulate the innate immune system via toll-like receptors (TLRs) [22]. This microbial dysbiosis, marked by an imbalance of protective and harmful microbes, compromises the intestinal barrier. Increased permeability allows the translocation of bacteria and dietary antigens into the underlying mucosa, triggering abnormal immune activation and perpetuating chronic inflammation [23]. For instance, emulsifiers and other components of processed foods common in the Western diet can exacerbate this process by destabilizing the mucus layer and promoting immune activation [24].

The Western dietary pattern’s role in the development of chronic diseases, such as IBD, is well established [25]. Several reviews and large cohort studies have consistently shown that the Western dietary pattern plays a significant role in the etiology of IBD [26,27,28,29,30]. These studies consistently highlight an increased risk of developing IBD among individuals with a high consumption of animal fats (omega-6 fatty acids), red and processed meat, sugar, refined grains, and ultra-processed foods [26,30]. In contrast, high fiber and fruit intakes are inversely associated with IBD risk [29]. The positive link between fat consumption, particularly trans fatty acids and omega-6 fatty acids, is most predominant in UC [31], while fish consumption is associated with a reduced risk of CD [32]. Western dietary patterns show an overall decrease in abundance in *Bifidobacterium*, *Lactobacillus*, and *Eubacterium*, while increasing the abundance of pathobionts such as *Clostridium bolteae*, *Ruminoccocus obeum*, *Ruminococcus gnavus*, and *Blautia hydrogenotrophica* [25]. The mucolytic nature of microbes combined with low fiber intake results in these bacteria using the mucus layer as their primary food source, leading to erosion of the epithelial barrier, gut permeability, and intestinal inflammation [33].

Adopting dietary patterns, such as the MD, has been shown to foster a health-associated microbiome and mitigate intestinal inflammation in patients with IBD [12,34]. The MD, rich in fiber, antioxidants, omega-3 fatty acids, polyphenols, and plant-based proteins, provides substrates for beneficial microbes, enhancing SCFA production and promoting a balanced gut microbiota and balancing inflammation [34]. Polyphenols, for example, exert prebiotic-like effects by selectively enriching commensal bacteria, while omega-3 fatty acids modulate inflammatory pathways by altering the composition of gut microbiota and reducing pro-inflammatory cytokines through the production of specialized pro-resolving mediators (SPMs) [35,36].

While preclinical evidence provides mechanistic insights on how the components of the MD influence intestinal epithelial barrier function and can alter immune function, clinical studies show moderate improvements in clinical markers, inflammation, and quality of life in IBD patients adhering to this diet [34]. These findings highlight the potential of the MD as a therapeutic dietary strategy. Additionally, dietary interventions can induce microbiota shifts, potentially resulting in significant and stable changes that contribute to improved disease outcomes [37]. However, substantial variation between studies underscores the need for further research into diet-microbiota interactions. Robust and consistent methodologies are essential to better understand these mechanisms, refine dietary strategies, and develop personalized treatment approaches for IBD patients.

## 4. The Mediterranean Diet in IBD: Transforming Clinical Biomarkers and Patient Outcomes

In the past five years, several studies have investigated the impact of dietary interventions, particularly the MD, in adults with IBD (Table 1). Chicco et al. (2020) conducted a six-month single-arm prospective MD intervention involving patients with UC (*n* = 84) or CD (*n* = 58) [38]. The study demonstrated significant improvements in obesity-related parameters, including body mass index (BMI) and waist circumference, and a reduction in liver steatosis assessed using abdominal ultrasound. Additionally, the intervention normalized C-reactive protein (CRP) and fecal calprotectin (FCP) levels, which are key biomarkers of inflammation and suggest reduced inflammatory burden, thereby improving disease control. Lewis et al. (2021) conducted a randomized control trial comparing the MD to the specific carbohydrate diet in patients with CD [13]. The study found no significant differences in remission rates or biomarker responses between the two diet interventions. The comparable symptomatic outcomes observed between the treatment groups may be attributed to similarities in the study diets, specifically that both were prepared using fresh ingredients. The study suggested that the MD may be preferable due to its greater ease of adherence and its broader health benefits, which extend beyond IBD management [13]. Haskey et al. (2023) examined MD versus a Canadian Habitual Diet in UC patients, with 40% of the MD group reporting improvement in the Simple Clinical Colitis Activity Index, maintenance or improvement of FCP, and alterations in microbiome composition [12]. These findings further emphasize the role of the MD in stabilizing inflammatory biomarkers and enhancing gut health, which are critical for maintaining disease remission and improving long-term outcomes. Dogan et al. (2024) investigated the MD, the MD with resveratrol supplementation, or the MD with curcumin supplementation in UC patients [39]. Improvements in waist/hip circumference, CRP, erythrocyte sedimentation rate, bowel movement frequency, MD adherence, and quality of life scores improved across all groups. However, supplementation with resveratrol or curcumin did not appear to amplify the effects of the MD. Although most studies have shown favorable results, Strauss et al. [40] and Zhang et al. [41] reported no significant changes in FCP and short-chain fatty acids in participants following an MD intervention.

Collectively, these studies highlight the potential of the MD to improve clinical outcomes and enhance quality of life in patients with IBD, with minimal reported adverse effects. The normalization of CRP and FCP levels across several trials underscores the diet’s ability to reduce systemic and intestinal inflammation, a critical goal in IBD management. These findings suggest that incorporating the MD as part of an integrated treatment approach could enhance disease control, improve patient outcomes, and potentially reduce the burden of pharmacologic therapies. However, the absence of control groups in some studies and the variability in study designs limit the ability to attribute these benefits solely to the MD. Rigorous, large-scale, randomized controlled trials with well-defined control diets are essential to validate these promising outcomes and to elucidate the precise mechanisms by which the MD exerts its therapeutic effects in IBD. Such research will further solidify the MD’s role as a cornerstone of holistic IBD management.

## 5. Mediterranean Diet Adherence: A Path to Better Health in IBD

The health benefits of the Mediterranean diet (MD) are well established. However, there are concerns regarding the successful adoption of its principles in regions outside of the Mediterranean. While potential barriers to the practical implementation of the MD in IBD are acknowledged, to our knowledge, no published reports specifically address these challenges. Despite these gaps, valuable insights can be gained by translating knowledge from other chronic conditions where similar barriers have been successfully addressed [42,43]. For example, limited understanding of the MD’s precise composition is common among patients and practitioners, highlighting the need for patient education on its specific components and associated health benefits. To improve acceptability, offering tasting sessions, food demonstrations, alternative meal ideas, and easy-to-prepare recipes could help.

Cultural identity plays a significant role in shaping food choices, often leading to resistance to adopting dietary patterns that diverge from cultural norms [44]. This presents important considerations for how the MD should be presented to non-Mediterranean populations. To facilitate its adoption, advice should be tailored to align with diverse cultural eating habits and traditional food views. Additionally, there is a perception that the MD is expensive. Providing budgeting tips and low-cost recipe ideas could help address financial concerns [43]. Furthermore, the availability of MD components in colder climates is often questioned, as the diet is traditionally associated with salads and fresh fruits. To address this, offering practical information on which foods are in season to purchase and providing alternative sources of fruits and vegetables (e.g., frozen or canned) will make the diet more feasible for individuals living in colder regions [42]. Concerns about weight gain from consuming olive oil and nuts are common, but research has shown that these components do not promote adiposity [45,46]. Education on the health benefits of replacing saturated fats with monounsaturated and polyunsaturated fats could help address these misconceptions.

While these factors—such as cultural identity, perceived costs, and ingredient availability—pose challenges to adopting the MD in IBD, recent studies demonstrate that high adherence to the MD is achievable within the IBD population (Table 2).

Papada et al. (2019) reported that higher adherence to the MD in patients with CD was associated with reduced disease activity (*p* < 0.001) and inflammation (*p* = 0.027) [47]. Similarly, Godny et al. (2020) observed that UC patients post-pouch surgery with higher MD adherence (*p* < 0.05) had lower FCP levels (*p* < 0.05) during an eight-year follow-up and lower rates of pouchitis [48]. Naqvi et al. (2021) reported a positive association between leafy green vegetables and a reduced FCP and an omega-6:omega-3 ratio of 8:1 was associated with normalized CRP. However, no specific relationships with MD adherence were observed [49]. Fiorindi et al. (2021) and Celik et al. (2023) found that higher MD adherence was associated with lower disease activity scores and improved mental health outcomes in both CD and UC [50,51]. A randomized trial by Haskey et al. (2022) demonstrated that a structured MD intervention in patients with UC improved diet quality (*p* = 0.007), and patients could successfully adhere to the MD [52]. Despite these promising findings, several limitations should be acknowledged. Studies have utilized varying symptom scoring systems, biomarkers, and MD adherence tools, which complicate direct comparisons, and the duration of dietary assessments varies widely. Additionally, the reliance on self-reported dietary data introduces potential inaccuracies, such as misreporting and bias, underscoring the need for future research in this area.

**Table 2:** Summary of research studies evaluating adherence to the Mediterranean diet and its impact on inflammatory bowel disease clinical aspects.

Author, Year	Population	Primary Objective	Diet Assessment Methods	MED Diet Assessment/Adherence	Results
Papada et al., 2019 [47]	Outpatient adults with endoscopically proven CD (*n* = 86)	Characterize the effects of MD adherence on quality of life, disease activity, and inflammatory markers	Assignment of MedDiet scores based on 24 h recall	MedDiet score evaluated by an experienced dietitian	↑ MedDiet scores in patients with inactive CD versus patients with active CD (*p* = 0.005) MedDiet score was negatively correlated with Harvey–Bradshaw Index (*p* < 0.001) and CRP (*p* = 0.027)
Godny et al., 2020 [48]	UC patients who underwent pouch surgery (*n* = 153)	Assess changes in inflammation markers, and reduced risk of pouchitis development in patients with UC after pouch surgery	Assessment of MD adherence during a 6-month interval between 2015 and 2018 based on a food frequency questionnaire	MED score Adherence defined as MED score ≥ 5	↔ MED scores between patients with active and inactive disease (*p* = 0.10) Patients with <200 mcg/g fecal calprotectin had ↑ MED score versus patients with elevated fecal calprotectin (*p* < 0.05) ↔ pouchitis development rates in patients with high MED diet adherence versus patients with low adherence (*p* = 0.17)
Naqvi et al., 2021 [49]	Adults (*n* = 66) with CD and clinical remission (steroid-free, clinical remission with Harvey–Bradshaw Index < 5 for > 3 months)	Assess the relationship between diet and markers of inflammation	A 3-day weighted food/drink intake, reviewed by a dietitian	pMDS score modified to exclude red wine consumption	Increasing daily servings of leafy green vegetables were associated with FCP ≤ 100 μg/mg (*p* < 0.05) omega-6:omega-3 polyunsaturated fatty acid ratio of 8:1 was associated with CRP ≤ 5 mg/L
Fiorindi et al., 2021 [50]	Adults with IBD (*n* = 62 CD, *n* = 18 UC)	Assess level of MD adherence in IBD patients with MEDI-LITE questionnaire	MEDI-LITE questionnaire conducted via face-to-face interview	MEDI-LITE questionnaire scores > 11 deemed adherent	↔ between CD and UC patients in the MEDI-LITE scores (*p* = 0.543) ↑ MEDI-LITE score in remission CD patients than active CD patients (*p* < 0.001) No significant differences in MEDI-LITE scores were found in remission UC patients and active UC patients with pouchitis (*p* = 0.218)
Haskey et al., 2022 [52]	Randomized controlled trial Adults (*n* = 28) with mild-moderate UC in remission (partial Mayo score 0–2)	Examining the proportion of participants achieving high adherence to the MD measured by the MDSSs Changes in diet quality, quality of life, nutritional diet adequacy were also measured as secondary analysis	Two intervention diets were used, the CHD (Canadian Habitual Diet) and the MD The MD group received sessions from dietitians to help adapt to the MD (based on the MD pyramid) with MD specific recipes, 4-week meal plan, food lists The CHD group followed their habitual diet	MDSSs (> 16 points) as measured after 12 weeks deemed adherent	After 12 weeks, there was a significantly higher MDSS in the MD intervention group compared to the CHD group (*p* = 0.010) and improved diet quality (*p* = 0.007) as measured by the Healthy Eating Index. No significance in changes in quality-of-life scores in both the groups
Celik et al., 2023 [51]	Adults diagnosed with IBD (*n* = 83; *n* = 38 UC patients; *n* = 45 CD patients)	Assess the effect of MD adherence on disease activity (Crohn’s disease activity index; Mayo Score for UC) and quality of life (Short Form-36) in IBD patients	Face-to-face interviews with a dietitian to provide MEDAS scores	MEDAS scores of ≤ 6, 7–9 and ≥ 9 categorized as low, acceptable and high adherence, respectively	Low MD adherence had higher Mayo Clinic scores (*p* = 0.018) No significant differences in Crohn’s disease activity index scores and BMI with MD adherence (*p* > 0.05) In UC patients, high MD adherence was associated with better scores in emotional problems (*p* = 0.03), mental health (*p* = 0.03), and overall health perception (*p* < 0.01) UC patients categorized as ’low adherence’ had higher UC Mayo Clinic scores (*p* = 0.018). In CD patients, MD adherence was not correlated with any sub-dimensions of quality of life measured by the Short Form-36 (*p* > 0.05).

↔: no difference; ↑: higher; CD: Crohn’s disease; IBD: Inflammatory bowel disease; MD: Mediterranean diet; UC: Ulcerative colitis; CD: Crohn’s disease; BMI: body mass index; MDSS: Mediterranean diet serving score; MEDAS: Mediterranean diet adherence score; pMDS: partial Mediterranean diet score.

## 6. Current Gaps in Research

Future studies should address existing limitations by standardizing tools for assessing dietary adherence and symptom scoring, enabling consistent comparisons across studies. The use of digital tools, such as mobile apps for real-time tracking of adherence and symptoms, could refine dietary strategies and improve patient outcomes. These tools can offer patients personalized guidance, track dietary intake, and provide real-time feedback, ultimately enhancing adherence to the MD and improving overall patient engagement. Incorporating control groups and conducting long-term, multicenter trials with larger sample sizes would provide valuable insights into the sustained benefits of MD adherence over time, helping to establish the MD as an effective long-term nutritional therapy for IBD management.

Consideration of cultural preferences, ingredient accessibility, and socioeconomic factors are necessary to develop tailored interventions that improve adherence to the MD. Structured dietary education programs, along with guidance on ingredient substitutions, could help overcome barriers to adoption. An examination of the impact of the MD on quality of life and mental health outcomes, particularly across subgroups with differing disease activity levels, is warranted.

Finally, advanced biomarker analysis and exploration of the mechanistic pathways underlying the MD’s anti-inflammatory effects would provide deeper insights into its therapeutic potential. It is equally important to focus on the variability of individual responses to the MD and the impact of confounding variables, as this will be key to advancing the field and optimizing personalized treatment strategies.

## 7. From Research to Practice: Bridging the Mediterranean Diet and IBD Care

**Figure 1:**
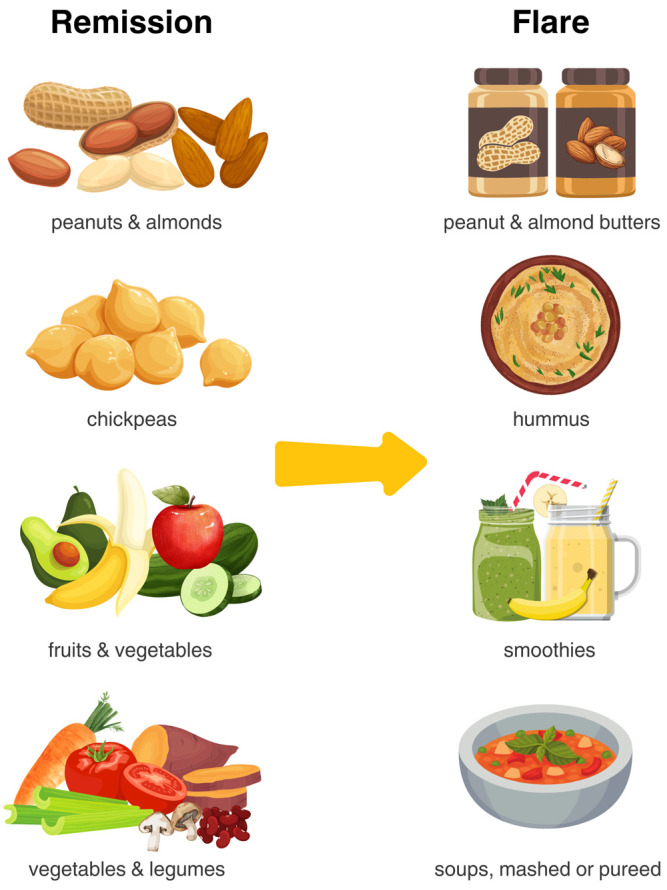
Visual comparison of dietary choices during remission versus active disease. This comparison highlights the practical implications tailored to different phases of IBD. During remission, patients can focus on nutrient-dense, whole foods such as nuts, legumes, fruits, and vegetables, consumed as tolerated. These choices support gut health, reduce inflammation, and promote overall well-being. During active disease, to ease digestion and minimize discomfort, the texture of foods can be modified—such as opting for cooked, peeled, or blended versions of fruits and vegetables and avoiding high-fiber, hard-to-digest items like nuts or raw legumes (Visual created by Leah D. D’Aloisio).

The literature highlights the effectiveness of a dietary intervention rooted in the core principles of the MD, as represented by the IBD Food Pyramid (Table 3) [53]. Research indicates that the MD is generally well tolerated among patients with IBD [52,54]. However, tolerance to specific foods can vary and may need to be adjusted based on the individual’s disease phenotype (Table 4 and Figure 1) [55].

Comorbid conditions, such as cardiovascular disease, colon cancer, diabetes, and living with overweightare rising in IBD and may require special consideration in treatment planning [56]. The presence of comorbidities often requires a more holistic approach to patient care, with physicians needing to balance the management of multiple health issues simultaneously. A multidisciplinary approach involving specialists from different fields is essential for optimizing patient outcomes. Managing IBD in the context of comorbidities highlights the increased complexity of treatment and the need for personalized care strategies to ensure both diseases are addressed effectively. Collaboration with a registered dietitian specializing in IBD can help patients tailor the MD to their needs, ensuring nutritional recommendations are appropriately implemented and nutrient deficiencies are avoided.

To address implementation challenges, we present key recommendations and strategies for counseling patients with IBD on adopting the MD more effectively. Affordability, availability, and personal dietary preferences should be considered, as these factors can significantly impact adherence in diverse patient populations. We acknowledge that these recommendations are primarily based on the expert opinion of our multidisciplinary team, which includes gastroenterologists, dietitians, and scientists specializing in gastrointestinal nutrition. To the best of our knowledge, no published manuscripts have comprehensively addressed these practical recommendations for patients.

(a) Choose Extra Virgin Olive Oil (EVOO)

Messaging: “Choose good fat, not low fat”

High-quality EVOO is the primary source of dietary fat in the MD, recognized as a functional food due to its nutritional value and health benefits [57]. The health benefits are largely attributed to its high oleic acid content (65–83% monounsaturated fatty acids) and bioactive compounds like tocopherols, polyphenols, and flavonoids [58]. EVOO is distinct from other common vegetable oils (e.g., sunflower, corn, or soybeanoils), which are rich in omega-6 fatty acids and have been associated with inflammation [59,60]. Additionally, the ratio of fatty acids in EVOO confers stability against oxidative thermal degradation, particularly by reducing the formation of volatile aldehydes compared to peanut and canola oils [61]. EVOO can be heated to as high as 400 °F (deep frying occurs at 350–375 °F). For those dishes that require prolonged heat (e.g., stir-frying), avocado oil is a better option.

Patient recommendations:

Using a high-quality EVOO is important, as lower-quality olive oils lose nearly all their beneficial properties. Look for a seal of approval from the International Olive Council or North America Olive Oil Association which certifies standards for olive oil’s purity and quality. High quality EVOO is typically packaged in dark bottles (amber, black, or green glass) to prevent oxidation caused by light and heat. To preserve quality, store the oil in a cool, dark place. For optimal flavor and nutritional benefits, consume the oil as soon as possible after the harvest date (generally within 18 months if unopened) and within 3 months once opened [62].

- Use EVOO liberally in cooking (stable to 420 °F) in place of omega-6-rich vegetable oils (e.g., sunflower, corn, soybean, palm, or canola oils).
- Drizzle EVOO on salads, vegetables, grilled fish, chicken, and pasta.
- Use EVOO as a base for salad dressings instead of commercially prepared salad dressings.
- Dip crusty bread in EVOO in place of butter or margarine.
- Add citrus-infused EVOO (e.g., lemon, orange) to breakfast smoothies, oatmeal, and yogurt.
- Add herb-infused EVOO (e.g., garlic, basil, rosemary) to salads, marinades and eggs.

(b) Fruit and Vegetables

Messaging: “The more colors the better, with fruit and vegetables being center stage”.

Fruit and vegetable consumption is a cornerstone of the MD, offering health benefits due to its rich fiber content, polyphenols, antioxidants, and micronutrients. Flavonoids are a type of polyphenol found in common fruits, vegetables, nuts, cocoa, tea, grains, and herbs. They are biologically active compounds responsible for the vibrant colors in fruits and vegetables [63]. Besides flavonoids, fruits and vegetables are key sources of fiber, potassium, folate, and antioxidants such as vitamin C, α-carotene, β-carotene, β-cryptoxanthin, and lycopene. Polyphenols are also crucial as immunonutrients due to their antioxidant and anti-inflammatory properties [64]. Research suggests that polyphenols commonly found in fruit and vegetables exert their effects primarily by remodeling the gut microbiota, acting as potential prebiotics that help shape a healthier microbial composition, strengthening barrier integrity, and modulating balanced immune responses [64].

Patient recommendations:

Fruits and vegetables are the foundation of meals. Varying the types of fruit and vegetables consumed throughout the day ensures a diverse intake of nutrients and compounds, each offering unique health benefits.

- Dark leafy greens can be used as salads, added to frittatas, eggs, smoothies, and soups.
- Add grated vegetables, such as carrots, zucchini, spinach, and kale to pasta sauces and soups.
- Canned tomato products are rich in lycopene (an antioxidant). A few tomato-centric recipes include shakshuka, stuffed vegetables, stews, curries, baked fish with tomatoes, and marinara sauce.
- Load up sandwiches with vegetables.
- Increase the nutritional value of smoothies by mixing in fruit and leafy greens.
- Top salads with fruit.
- Add fruit to yogurt or cereal.
- Try baked fruit topped with oatmeal, cinnamon, and maple syrup for dessert.
- Roast vegetables to increase flavor, drizzle with olive oil.
- To save preparation time, consider packaged ready-to-eat fresh fruit and vegetables. Frozen and canned fruit and vegetables are budget-friendly options.
- Choose canned vegetables packed in water and look for “no salt added” or “low sodium” options with no added sugar, preservatives. or artificial additives. Even when purchasing “no salt added” options, it is good practice to rinse them under water to remove any additives or preservatives.
- Choose canned fruits packed in water to reduce the sugar content. Whole fruits (e.g., peaches, pears, etc.,) have generally fewer additives than “cocktails”. Check for extra additives, as some products labeled “no sugar” may still contain artificial sweeteners. It is good practice to rinse them under water to remove extra sugar and preservatives.

(c) Whole Grains and Starchy Foods

Messaging: “Feed your microbes fiber, or they will feed on you”.

Whole grains are essential to the MD as they are rich in vitamins, minerals, lignans, phytochemicals (phenolic acids, polyphenols, and phytosterol compounds), and fiber [65,66]. Common examples of grains include whole wheat, brown rice, oats, millets, barley, and rye. Another form of fiber is resistant starch. Resistant starch is a broad category of structurally complex starches resistant to digestive enzymes in the gastrointestinal tract, commonly found in green bananas, unprocessed whole grains, legumes, and cooked then cooled rice, pasta, or potatoes [67]. Many patients believe that they should avoid dietary fiber. Still, strong evidence suggests that dietary fiber can positively impact the gut microbiome, improve IBD symptoms, balance inflammation, and improve health-related quality of life [68]. Currently, there are no specific guidelines on the relative proportions of different dietary types of fiber to include in the diet. For patients who experience difficulty tolerating fiber or they have CD with strictures (intestinal narrowing), fiber intake can be adjusted by modifying the food textures, such as blending, mashing, or pureeing to enhance digestibility.

Patient recommendations:

- Breakfast is one of the easiest ways to increase fiber by consuming whole-grain toast or oatmeal.
- Swap refined grains like white bread, white rice, and pasta for whole grains like brown rice, quinoa, bulgur, barley, and farro.
- Add barley to soups to boost soluble fiber.
- Psyllium can be sprinkled on food.
- Cook, cool, reheat pasta, rice, and potatoes to increase resistant starch.
- Use whole grain flours in baking (e.g., oat flour).

(d) Nuts and Seeds

Messaging: “Embrace nut and seed butters”.

The MD recommends consuming nuts and seeds daily due to their high nutrient density, including unsaturated fats, protein, fiber, and polyphenols. Nut consumption is also associated with several other health benefits. For example, a recent systematic review indicates that consuming 28 g of nuts daily is associated with a reduced risk of cardiovascular disease, cancer, and overall mortality [69]. Given that patients with IBD are at risk for comorbid conditions (e.g., cardiovascular disease, colon cancer, diabetes, living withoverweights), it is imperative for people with IBD to adhere to healthy eating guidelines to prevent further deterioration in health. Additionally, the enrichment of butyrate-producing gut bacteria from nut consumption supports the hypothesis that nuts have a prebiotic effect [70]. In patients with Crohn’s disease and ulcerative colitis, dietary patterns before the onset of the disease often show a decreased intake of nuts and seeds, highlighting the potential importance of emphasizing the consumption of foods for supporting gut health [71].

Whole nuts and seeds can be problematic in certain situations for individuals with IBD and may need to be consumed in a smooth nut or seed butter form. During active flare-ups, nuts and seeds may worsen intestinal symptoms such as pain, bloating, and stool frequency. In cases of stricturing disease, consuming nuts and seeds in their whole form can increase the risk of blockages. Therefore, in these patients and those who have had a recent IBD-related luminal surgery, it is recommended to enjoy these foods in their smooth butter form to prevent worsened symptoms or disease complications.

Patient recommendations:

- Choose nuts higher in monounsaturated fats such as almonds, cashews, macadamia, hazelnuts, pistachios, pecans, and walnuts.
- Nuts and seeds can be consumed as nut butter for easier digestion and improved tolerance. This should be favored in those with active disease, recent luminal surgery, and those with known intestinal strictures.
- Opt for raw, unsalted nuts or nut butters without added sugars, salt, or fats.
- A handful of raw nuts makes a healthy, nutrient-rich alternative to processed snacks.
- Tahini (ground sesame seeds) is versatile and can be used in sauces, dressings, or drizzled over roasted vegetables or grain bowls to enhance flavor.
- Add nuts and seeds to enhance dishes like yogurt, smoothies, oatmeal, or fruit.
- Chia seeds expand when moistened, making them ideal for creating jams and puddings.
- Soaking most nuts can improve their digestibility, reduce phytic acid, and enhance nutrient absorption. Soak most nuts for 4 to 12 h, or overnight, to improve their digestibility. Softer nuts, such as cashews, require a shorter soaking time, while harder nuts, like almonds, may benefit from a longer soaking period for optimal results.

(e) Legumes

Messaging: “Add legumes gradually”.

Legumes are valuable protein and soluble fiber sources and contain bioactive compounds, including phytochemicals with known antitumor properties [72]. Systematic reviews and meta-analyses of prospective cohort studies have shown moderate-quality evidence that consuming legumes at a frequency of at least four 100 g servings per week can aid in preventing cardiometabolic risk factors and colorectal cancer [73]. Additionally, individuals with IBD often have an inadequate intake of legumes [74].

Patient recommendations:

- To cook dried beans, use a 1:4 ratio of beans to water. Soak beans overnight to reduce lectins, which can interfere with nutrient absorption and cause discomfort. Discard the soaking water, rinse the beans, and cook in fresh water. Boil for 10–30 min at high heat to deactivate most lectins. Avoid slow cooking or eating raw beans, as they may not reduce lectins effectively.
- Look for canned beans labeled low-sodium or with no salt. Rinse before serving or cooking to remove sodium that is added during processing. Rinsing canned beans can help make them more digestible.
- Lentils may be easier to digest than other starchier legumes like black beans or chickpeas, so start with lentils if other legumes cause too much digestive distress,
- Add legumes to the diet gradually—start with 2 to 4 tablespoons of beans or lentils at a time, then increase intake as the body adjusts.
- Legumes lend themselves to soups, tacos, burritos, and chili, though you can also eat them independently.
- Toss them on top of salads, purée them into a bean dip, or use them as a meat substitute in burgers, stews, and soups.
- Beans can be roasted and used as snacks and salad toppers.

(f) Dairy Products

Messaging: “Rethinking dairy on the MD”,

Dairy consumption in IBD has been controversial, with ongoing debate about whether patients with IBD should avoid milk and dairy products. Cross-sectional studies have demonstrated that the rate of intolerance to dairy among IBD patients is similar to the general population [75,76]. Moreover, a growing body of evidence indicates that dairy food consumption does not increase inflammatory biomarkers, with multiple studies documenting significant anti-inflammatory effects [75]. Dairy products are important nutritional sources that provide more calcium, protein, magnesium, potassium, zinc, and phosphorus per calorie than any other typical food in the adult diet.

Patient recommendations:

- Replace heavy cream and processed cheese, instead, choose fermented cheeses like feta, Brie, cotija, Swiss, halloumi, ricotta, Manchego, and Parmesan.
- Include fermented dairy (e.g., plain Greek yogurt, kefir) and limit flavored yogurts that tend to be higher in sugar, add flavorings (lemon, maple syrup, berry purees) to sweeten if needed.
- Yogurt, kefir, and aged hard cheeses are lower-lactose options.
- Trial lactose-free options, smaller portions spread throughout the day, if tolerance is an issue.

(g) Fatty Fish, Eggs, White Meat, and Red Meat

Messaging: “Rethink our perspective on protein”.

In contrast to the Western diet, which is high in red and processed meats and linked to an increased risk of developing IBD [77], the MD emphasizes fish and shellfish as primary protein sources [40]. Although studies on the association between fish consumption and IBD risk show inconsistent results, an inverse relationship has been observed between fish intake and the risk of CD [32]. Additionally, a negative association has been noted between the consumption of omega-3 fatty acids and the incidence of UC [32]. Although the role of omega-3 fatty acids in IBD remains debated, consuming fatty fish such as salmon, mackerel, sardines, tuna, and herring—rich in omega-3 fatty acids—may provide a protective benefit against IBD [78]. Fish is also considered highly nutritious, offering antioxidant, anti-inflammatory, wound-healing, neuroprotective, cardioprotective, and hepatoprotective properties [79]. To date, there is no evidence linking egg consumption with IBD.

Patient recommendations:

- Choose white meats (poultry without skin) instead of red meats, pork, or processed meats, sausages, cold meat, or paté.
- Enjoy omega-3-rich fish such as tuna, sardines, and salmon, either fresh or canned.
- Consume red meats (lamb, mutton, beef, pork, veal, goat, horse) less frequently. Opt for lean cuts and prepare in stews, stir-fries, or soups.
- Limit intake of smoked, salted, and processed meats.
- Eggs can be enjoyed daily and are often a well tolerated protein source.
- Aim for moderate portions of 4 ounces per meal.

(h) Ultra-processed Foods, Sweets, and Alcohol

Messaging: “Choose minimally processed foods for better health”.

Ultra-processed foods and sweets are not regularly consumed as part of the MD. Ultra-processed foods such as soft drinks, packaged snacks (sweet and savory), reconstituted meat products, and pre-prepared frozen meals primarily contain food additives, leaving little of the natural food [80]. These products are often high in saturated and trans fats, salt, high-fructose corn syrup, emulsifiers, artificial colors, and added sugars while low in phytochemicals, protein, micronutrients, and fiber [80]. Increased consumption of ultra-processed foods is associated with higher risk of IBD, with a dose–response relationship indicating a greater risk of disease flares in CD [81]. Alcohol intake has not been identified as a risk factor in IBD; however, some patients report a subjective worsening of symptoms, and some studies reporting worsening of disease activity with greater alcohol intake [82].

Patient recommendations:

- Reduce the consumption of packaged, processed foods, and commercial sauces containing maltodextrin, carrageenan, carboxymethylcellulose, polysorbate-80, titanium dioxide, sulfites, and xanthan gum.
- Limit sugary beverages and artificial sweeteners (e.g., aspartame, sucralose, saccharin). Replace soda and juices with water.
- Coffee, tea, and herbal infusions (rich in flavonoids) are allowed, but they should be consumed preferably without any sweetener.
- Avoid high-fat and sugar pastries, industrial bakery products (e.g., cakes, donuts, or cookies), and industrial desserts (e.g., puddings, custard). Save cakes and sweets for special occasions
- Use herbs, spices, garlic, and onions to increase food palatability and reduce the use of salt in cooking.
- Limit to low-risk alcohol consumption. Patients with IBD often report worse gastrointestinal symptoms following alcohol consumption. In the available literature, alcohol use in patients with IBD trends toward harmful effects; however, more research is needed [83].

(i) Modifying the Mediterranean diet according to the stage of the disease.

Messaging: “Tailor the Mediterranean diet to your disease stage for maximum benefit—nourish your body with the right foods at the right time!”

Certain dietary components may be challenging for some individuals to tolerate, but this varies from person to person and does not apply to everyone. All patients should be encouraged to personalize their diet to their disease stage (Table 4). For patients with strictures, limiting fibrous foods and insoluble fiber may be beneficial in reducing the risk of blockage [55,84].

## 8. Conclusions

In conclusion, the MD holds promise as an adjunctive approach to managing IBD. Mounting evidence supports its positive impact on clinical biomarkers and patient outcomes. Its practical nature, especially in terms of its feasibility for integration into clinical settings, makes it an attractive option for patients and healthcare providers. As a flexible, inclusive, nutrient-rich diet, the MD promotes the consumption of health-promoting foods like fruits, vegetables, whole grains, and healthy fats. This enhances patient adherence, especially compared to other more restrictive dietary approaches, such as low FODMAP or exclusive enteral nutrition, thus enhancing its effectiveness in everyday clinical practice.

Multidisciplinary teams, particularly dietitians, play a pivotal role in optimizing dietary interventions for patients with IBD. Registered dietitians (RDs) are essential for guiding patients in adopting the MD, ensuring cultural consideration and nutritional adequacy. Through proper education and support, dietitians can help manage potential challenges related to the diet’s integration into treatment plans, monitor progress, and adjust strategies as necessary.

Further research is needed to deepen our understanding of the underlying mechanisms of the MD and to optimize its application in personalized nutrition treatment strategies.

## Figures and Tables

**Table 1:** Intervention studies that assess the effect of the Mediterranean diet on clinical biomarkers in inflammatory bowel disease.

Author, Year	Study Design	Population	Diet Intervention	Outcomes	Results	Limitations
Chicco et al., 2020 [38]	Single arm intervention study	*n* = 142 adults with active IBD (*n* = 84 UC; *n* = 58 CD) Disease activity assessed through the Crohn’s disease activity index for CD and partial Mayo score for UC	Six months of MD adherence Nutritional counseling provided by a nutritionist: ≥2 vegetable servings per meal, 1–2 fruit servings per meal, and 1–2 bread/cereals servings per meal, and olive oil at every meal alongside ≥2 legume servings weekly, ≥2 fish/seafood servings weekly, 2–4 egg servings weekly, and 2 poultry servings weekly and 2 dairy foods servings daily while limiting red meat and sweets to <2 servings per week. Patient adherence was assessed using 24 h recall during nutritional interviews after 6 months	Nutritional status, presence/severity of liver steatosis, therapy response Anthropometric measures: weight, BMI, visceral fat, lean body mass, fat body mass, waist circumference (measured with bioelectrical impedance analysis) Serum lipid profile and lipid function (chemical analysis) Hepatic steatosis (abdominal ultrasound exam) Quality of life (Inflammatory Bowel Disease Questionnaire)	In UC patients: ↓ BMI (*p* = 0.002) ↓ Waist circumference (*p* = 0.037) ↓ N of patients with elevated CRP (*p* = 0.013) ↓ N of patients with FCP > 250 mg/kg (*p* = 0.049) ↑ Quality of life (*p* < 0.001) In CD patients: ↓ BMI (*p* = 0.023) ↓ Waist circumference (*p* = 0.040) ↓ N of patients with elevated CRP (*p* = 0.035) ↓ N of patients with FCP > 250 mg/kg (*p* = 0.035) ↑ Quality of life (*p* < 0.001)	The lack of a control group, therefore improvements may have occurred independent of the diet. Participants were in clinical remission or affected by mild disease which might lead to overestimated effect of the dietary intervention on disease activity and QoL. Researchers did not use any specific score to quantify adherence to diet, and this was mainly based on patients’ dietary recall.
Zhang et al., 2020 [41]	Randomized controlled trial	Adults (*n* = 40) with luminal CD in remission (Harvey Bradshaw Index < 5)	Two diet groups were studied: Patients that habitually consume a diversified diet pattern [DD] [higher plant-based and lower red and processed meat-based diet] compared to patients following a non-diversified diet pattern [NDD] + MD intervention for 12 weeks Adherence was assessed by 3-day weighted food records	To compare microbiota composition and function between patients in the DD group with patients in the NDD group following a 12-week structured dietary intervention based on principles from the MD	No difference in microbial beta-diversity between the two groups was observed (*p* = 0.43) The NDD + MD group demonstrated an increase in *Faecalibacterium*. No association of diet with fecal SCFAs or FCP.	No significant changes in FCP levels observed at week 12, likely due to the clinically and biochemically quiescent baseline disease state. Researchers did not use any specific score to quantify adherence to diet, and this was mainly based on patients’ dietary recall.
Lewis et al., 2021 [13]	Randomized control trial	Adults (*n* = 93, 63% women) with active CD with mild to moderate CD symptoms (short CD Activity Index Score > 175 and < 400)	Participants randomly received either the SCD or the MD for the first 6 weeks (prepared meals consisting of breakfast, lunch, dinner, and 2 snacks) After the first 6 weeks, participants were instructed on food purchase and preparation that aligned with MD Participants completed a 24 h recall at baseline, weeks 6 and 12. These data were used to assign an alternate MD score	Primary outcome: Symptomatic remission at week 6 without increasing CD medication Secondary outcome: changes in FCP and CRP	Symptomatic remission in participants at week 6 (*p* = 0.77) and week 12 (*p* = 0.87) was not superior in SCD as compared to MD Among those with an elevated FCP at screening, FCP response was achieved in 8/23 participants (34.8%) with SCD and 4/13 participants (30.8%) with MD (*p* = 0.83) Among those with elevated CRP at screening, CRP response was achieved in only 2/37 participants (5.4%) with SCD and 1/28 participant (3.6%) with MD (*p* = 0.68) from screening to week 6	The study was not designed to assess endoscopic healing. Symptomatic remission was common, few patients achieved combined symptomatic remission and resolution of inflammation. This study included patients with longstanding disease, many of whom had been treated with biologics, which limits generalizability.
Haskey et al., 2023 [12]	Randomized controlled trial	Adults (n = 28) with mild-moderate UC in remission (partial Mayo score 0–2)	Two intervention diets were used, the Canadian Habitual Diet and the MD, for 12 weeks The MD group received sessions from dietitians to help adapt to the MD (based on the MD pyramid) The Canadian Habitual Diet group followed their habitual diet MD adherence was assessed using the MD serving score (MDSS)	Assessing whether MD intervention could reduce SCCAI, FCP levels, and microbiome changes	40% of MD intervention group reported minor improvements in the SCCAI scores, 27% achieved clinical response, whereas 1% reported a decrease in 1-point SCCAI score At week 12, 75% [9/12] of participants in the CHD had an FCP > 100 μg/g vs. 20% [3/15] of participants in the MD group The MD induced alterations in microbial species known to be protective in UC (*Alistipes finegoldii* and *Flavonifractor plautii*), as well as the production of short-chain fatty acids (*Ruminococcus bromii*)	A 12-week follow-up may limit insights into long-term MD effects on disease activity. Results are not generalizable to active IBD patients, as only adults in clinical remission were studied. The small sample size may reduce the study’s statistical power.
Strauss and Haskey et al., 2023 [40]	Randomized open-label trial	Adults (*n* = 40) with active UC (partial Mayo score > 2)	Participants were assigned to MD + low sulfur diet or habitual diet Adherence assessed by the MDS	Improvement in total Mayo score and partial Mayo score	No changes in MDS or FCP were observed within or between groups Marginal improvements in partial Mayo score (median 2.0) were observed from baseline and week 8 in participants following the intervention diet (*p* = 0.003); however, this also occurred in the habitual diet (*p* = 0.007) Valerate (SCFA) and glycochenodeoxycholic acid (bile acid) were significantly different between groups at baseline and week 8 (*p* = 0.05 and *p* = 0.02, respectively)	Despite a significant decrease in sulfur intake in the MD intervention from baseline to week 8, this did not translate into a reduced FCP. The study sample was heterogeneous in disease activity, reflected by the wide range of partial Mayo scores and total Mayo scores at baseline. Baseline MDS did not differ between intervention groups, nor did it change over time within the intervention group, underscoring the need to assign participants to dietary interventions distinct from their baseline diet.
Dogan et al., 2024 [39]	Three-arm intervention study	Adults (n = 46) with mild-to-moderate UC determined by a gastroenterologist	Participants were randomly assigned into three groups: MD, MD + resveratrol (1600 mg/day), MD + curcumin (500 mg/day) for 8 weeks Bi-weekly MD education with dietitian Patient adherence was assessed using the MD adherence scale (MEDAS) with 14 items scored as either 0 or 1	Truelove–Witts Index of disease activity, serum inflammatory markers, and quality of life (measured by Short Form-36)	Significant improvement post intervention was observed within groups for waist and hip circumference, bowel movements, CRP, erythrocyte sedimentation rate and an increase in quality-of-life scores (*p* < 0.05)	Absence of clinical biomarkers (e.g., fecal calprotectin, cytokine data, and endoscopic imaging). The study was limited to individuals with mild-to-moderate active disease, which restricts the generalizability of the findings to individuals in remission or with severe active disease.

Abbreviations: ↑: higher; ↓: lower; IBD: inflammatory bowel disease; UC: Ulcerative colitis; CD: Crohn’s disease; MD: Mediterranean diet; BMI: body mass index; CRP: C-reactive Protein; FCP: Fecal calprotectin; SCD: Specific carbohydrate diet; SCCAI: Simple Clinical Colitis Activity Index.

**Table 3:** Key features of the inflammatory bowel disease food pyramid.

Guiding Principles			
▪ Main meals consumed daily should include three components: vegetables, fruits and whole-grains. In addition, legumes, fermented dairy should be consumed, though not necessarily in every meal. ▪ Stock kitchens with minimally processed foods. ▪ Eating 3–5 x/day, smaller meals may be better tolerated when gastrointestinal symptoms are present. In some patients, structured fasting can help. ▪ Frequencies and serving sizes should be aligned with the individual’s energy requirements. ▪ Mindful eating, thorough chewing, and pausing for meals are essential for individuals with IBD. Since digestion begins in the mouth, paying extra attention to these habits becomes even more crucial when the gut is inflamed to optimize digestive and absorptive functions. ▪ Slowly adapt your diet to make it more MD-like, pick one change every week and incorporate it gradually.			
Every Meal			
	**Frequency**	**Serving Size**	Included Foods *
Extra Virgin Olive Oil	1 serving/main meal	1 tablespoon	High quality oil (see commentary about choosing quality)
Fruit	1–2 servings/main meal	½ cup or 1 medium sized piece	A variety of colors in both vegetables and fruits is strongly recommended to ensure intake of a broad range of micronutrients and phytochemicals
Vegetables	2 servings/day plus 1–2 servings/day of leafy greens	½ cup or 1 medium sized piece plus 1 cup raw	
Cereals	1–2 servings/main meal	1 cup cooked or 1 slice of bread	Includes bread, pasta, rice, oats Preferably whole grains as tolerated
**Daily**			
Starchy Foods (Resistant Starch)	1–2 servings/day	1 cup per day	Includes cooked, cooled reheated rice, pasta, potatoes, winter squash, yams, cassava, and taro
Dairy	2 servings/day	¾ cup yogurt or 1.5 ounces of hard cheese (cheddar) or 1 cup of milk	Yogurt (Greek yogurt, low sugar), kefir or hard cheese may be better tolerated due to lower lactose content
Nuts/Seeds	1–2 servings/day	1 ounce or 1/4 of a cup	Without sugar, fat or salt, nut/seed butters may be better tolerated
**Weekly**			
Legumes	3 servings/week	¾ cup (150 g) cooked	Includes beans, peas, lentils, edamame, and soy
Fatty Fish and Seafood	2 servings/week	6 ounces twice per week	Includes salmon, mackerel, tuna, trout, herring, and sardines
Eggs	1 egg/daily	1 large egg (with yolk and white)	Whole eggs, including those used for cooking and baking
White Meat	2 servings/week	4 ounces	Includes skinless chicken and turkey Choose lean poultry (e.g., breast, wing, or back portions)
Red Meat	1 serving/week	< 8 ounces per week	Includes pork, beef, and lamb
**Limit**			
Sweets	< 2 servings per week		Includes sugar, candies, pastries, sweetened fruit juice, and soft drinks Fruit should be eaten in place of sweets
Processed Meat	< 1 ounce (30 g) per week		Includes deli meats, ham, sausages, bacon, jerky, and hot dogs
Ultra-Processed Foods	Avoid as much as possible		Includes ice cream, chips/crisps, mass-produced bread and bread products, crackers, biscuits, cookies, instant soups
Additives	Limit		Includes maltodextrin, carrageenan, carboxymethylcellulose, polysorbate-80, titanium dioxide and sulfites, xanthan gum, aspartame, sucralose, saccharin
Alcohol (includes spirits, beer and wine)	Limit		Replace with water or herbal infusions

* Does not apply to patients with strictures, texture modification is needed.

**Table 4:** Modifications of fruit and vegetables based on the stage of disease.

	Active	Strictures/Ileostomy ^#^	Remission
Fruit	Remove skin/peel Blend into smoothies Apples, bananas and canned/pureed fruit packed in water or juice Pureed fruit (e.g., applesauce, fruit coulis) Cooked/stewed fruit * Limit: dried fruit, coconut, pineapple, prunes	Follow active disease recommendations Smoothies are a great option	No restrictions, based on individual tolerance
Vegetables	Cook vegetables until fork tender and remove peels Blend greens into smoothies Consider blended soups * Limit: brussels sprouts, cabbage, cauliflower, kale, asparagus, peas, corn, artichoke	Follow active disease recommendations **plus:** Avoid skins, tough stalks and seeds as well as raw salads	No restrictions, based on individual tolerance
Whole Grains and Starchy Foods (Resistant Starch)	Focus on including soluble fiber: barley, oats, psyllium Green bananas Cook, cool, reheat pasta, rice, sweet potato, and potatoes Limit whole wheat flour, wheat bran	Avoid insoluble fiber, corn hulls, popcorn, wild rice Cook, cool, reheat pasta, rice, and potatoes	Replace refined grains with whole grains, including both insoluble and soluble fiber Cook, cool, reheat pasta, rice, and potatoes No restrictions, based on individual tolerance
Nuts and Seeds	Nut and seed butters without added sugar, salt, or fat	Ground nut and seed butters without added sugar, salt, or fat	No restrictions, based on individual tolerance
Legumes	Lentils, split pea, tempeh or tofu	Mashed or pureed beans (e.g., hummus) or tofu	No restrictions, based on individual tolerance
Dairy Products	Lower lactose, lactose-free or fermented options may be better tolerated	No restrictions, based on individual tolerance	No restrictions, based on individual tolerance
Fatty Fish, Eggs, White Meat, and Red Meat	Focus on fish, skinless poultry and eggs while limiting red meat	Stewed, fork tender meat Avoid tougher cuts of meat, unless slow-cooking or stewing (e.g., chuck, brisket, or round, chicken wings), sausages with casing.	No restrictions, based on individual tolerance

^#^ Strictures are narrowing in the intestine. * Based on individual tolerance as tolerance may vary.
